# Host-Seeking Behavior in the Bed Bug, *Cimex lectularius*

**DOI:** 10.3390/insects2010022

**Published:** 2011-03-07

**Authors:** James T. Suchy, Vernard R. Lewis

**Affiliations:** 1 Department of Public Health, University of California, Berkeley, CA 94720, USA; 2 Department of Environmental Science, Policy and Management, University of California, Berkeley, CA 94720, USA; E-Mail: urbanpests@berkeley.edu

**Keywords:** *Cimex lectularius*, bed bug, host-seeking, behavior, movement, time-lapse photography

## Abstract

The reemergence of the bed bug, *Cimex lectularius* Linnaeus, has recently spawned a frenzy of public, media, and academic attention. In response to the growing rate of infestation, considerable work has been focused on identifying the various host cues utilized by the bed bug in search of a meal. Most of these behavioral studies examine movement within a confined environment, such as a Petri dish. This has prevented a more complete understanding of the insect's host-seeking process. This work describes a novel method for studying host-seeking behavior, using various movement parameters, in a time-lapse photography system. With the use of human breath as an attractant, we qualitatively and quantitatively assessed how bed bugs navigate their environment between its harborage and the host. Levels of behavioral activity varied dramatically between bed bugs in the presence and absence of host odor. Bed bugs demonstrated not simply activation, but attraction to the chemical components of breath. Localized, stop-start host-seeking behavior or alternating periods of movement and pause were observed among bed bugs placed in the environment void of human breath, while those exposed to human breath demonstrated long range, stop-start host-seeking behavior. A more comprehensive understanding of bed bug host-seeking can lead to the development of traps and monitors that account for unique subtleties in their behavior. The time-lapse photography system uses a large, artificial environment and could also be employed to study other aspects of the insect's behavioral patterns.

## 1. Introduction

Over the last decade, a pest that faded into the obscurity of childhood rhyme has reentered the global consciousness. Heightened pesticide resistance, cheaper international travel, the transfer of second hand furniture and clothing, and lack of public knowledge are proposed explanations for the dramatic resurgence of the bed bug, *Cimex lectularius* L. (Heteroptera: Cimicidae) [1]. Their increasing prevalence in anthropogenic locations throughout Europe, North America, Asia, and Australia [2–4] suggests a long and extensive occupation.

The ectoparasitic bed bug spends the majority its life aggregated in refuges, hidden from plain sight [1]. Thigmotropism [5,6] and pheromonal chemotropism [7,8] encourage inactivity in clustered conspecifics. Despite possessing a predominantly sedentary nature, bed bugs' success and public infamy are attributed to their rapid movement into neighboring rooms and apartments [9,10]. This spread is due to active movement by the bed bug itself, as a result of pesticide disturbance [11], intersexual conflict [12], host stimuli [13], and other triggering forces. Passive movement, such as accidental host transport [6], may also facilitate spread of the bed bug. Few studies have examined bed bug movement ecology [14], although such information is important to understanding the widening distribution of this pest.

Of particular neglect is examination of the kineses and taxes associated with bed bug host-seeking, in which host stimuli influences movement. Vertebrates produce a variety of visual, mechanical, chemical, and thermal cues that are detectable to the hematophagous insect [15]. These cues formulate a sensory ‘pathway,’ guiding the insect's movement indirectly or directly towards the host. For the bed bug, a blood meal is necessary for survival, development, and proliferation [1]. This combination of obligatory hematophagy and isolation from the host reflects the large degree to which host-seeking influences the bed bug's movement. Bed bugs are reported to travel distances of 6 or more meters, to reach a host [6]. Some recent studies have begun to study bed bug host-seeking in relation to certain host-derived metabolic byproducts, such as CO_2_, heat, octenol, and lactic acid [16,17]. However, conclusions are based on dichotomous measures, evaluating behavioral response through presence or absence in a trap, harborage, or area of a container [16–18]. Such results don't clearly demonstrate the movement patterns used by the host-seeking bed bug to traverse the environment between the harborage and the host. Although useful for development of traps and monitors, this methodological bias still leaves a gap in our understanding of host-seeking behavior and the role host-seeking plays in the bed bug's movement. By better understanding the interaction between the insect, the host, and the environment, more successful and cost-effective integrated pest management (IPM) strategies, reflecting a comprehension of their movement ecology, could be devised.

Rivnay [19] was among the first to study host-seeking bed bug movement, in response to potential host cues (blood, temperature, sweat, sebum, bile). While informative at the time, such early movement studies were limited to descriptive observation and line tracings to track the animal's path. Techniques used to study animal movement have changed dramatically since then. With the advent and growing affordability of computers and digital cameras, modern methods allow the researcher to study animal movement in a more precise, thorough, and reproducible way. We felt the resurgence and limited understanding of the bed bug justified a reexamination of the insect's host-seeking movement, using contemporary, experimental methods.

The goal of this work was to present a qualitative and quantitative study of the host-seeking behavior of starved, adult *C. lectularius*. Behavioral response to the presence or absence of long-range olfactory cues in the immediate surroundings was quantifiably examined using a variety of parameters describing the insect's movement. We illustrate the searching behaviors of starved males and females in both of these environmental conditions. To the best of our knowledge, this is the first time modern technology has been used to track the precise movement patterns of the host-seeking bed bug throughout a semi-natural environment.

## 2. Materials and Methods

### 2.1. Bed Bugs

All *Cimex lectularius* came from a laboratory strain, grown by Harold Harlan (Crownsville, MD), who has sustained this strain for more than 30 years through direct feeding on himself. Bed bugs were not fed while in our possession. Bed bugs of all stages were kept in a circular, glass container that was covered by a large watch glass. Pieces of folded cardboard served as refuge inside of the container. The container was kept inside of an incubator (Barnstead, Lab-Line No.152) that was maintained at a steady 30 °C and an L14:D10 photoperiod. Featherweight soft-tipped forceps (BioQuip Products, Rancho Dominguez, CA) were used to remove bugs from the container, for experimental testing. Bed bugs in the experiment had been starved of blood for three weeks.

### 2.2. Arena Design

**Figure 1:**
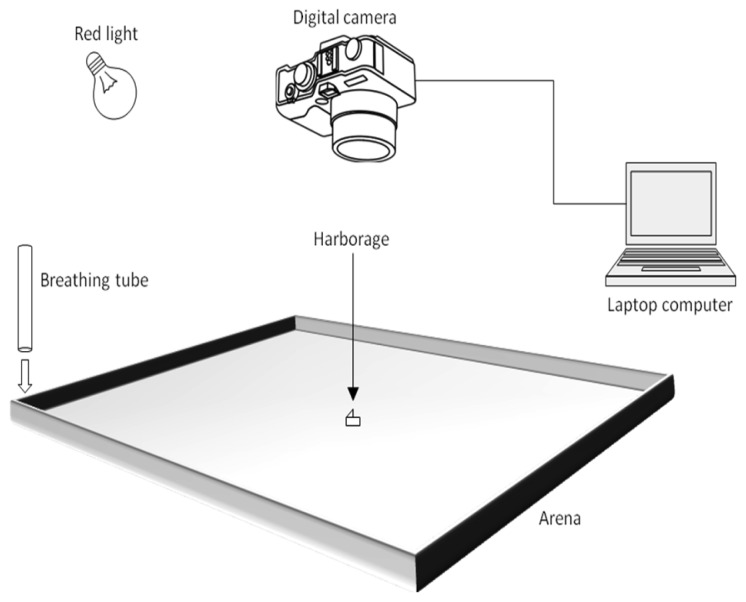
The time-lapse photography system and other equipment used to track the behavioral movement of the bed bugs throughout the experimental arena.

A custom-made, white, acrylic tray (Nisei Plastics, Oakland, CA) was used as the experimental arena (Figure 1). The arena had a square base (91.44 × 91.44 cm) and short sides (5.08 cm). The sides of the arena were layered with Insect-A-Slip (BioQuip Products, Rancho Dominguez, CA), to prevent the bed bugs from escaping. The bottom of the arena was covered with a sheet of butcher paper (90.17 × 90.17 cm). White masking tape was used to hold down the butcher paper and keep the bugs from crawling underneath. The center of the arena was measured and marked lightly with pencil on the butcher paper. After each replicate, the used butcher paper was discarded and the arena was wiped down with 70% ethanol, before being reused.

### 2.3. Time-Lapse System

A Canon PowerShot SX110 IS digital camera (Canon, NY, USA) was positioned to look directly down, into the arena (Figure 1). The camera was held above the arena (147.32 cm) by a freestanding U-frame structure, built with ‘2 by 4’ (5.08 × 10.16 cm) wood. The U-frame was composed of a horizontal cross-beam (3.66 m), held above the ground by two ‘legs’ (1.52 m). Two ‘feet’ (0.61 m) were attached to the bottom of each ‘leg,’ giving the structure stability. An adjustable speaker mount was screwed to the face of the cross-beam, and the camera was attached to the speaker mount. The camera was connected to USB cables (5.79 m) running to a separate room and plugged into a laptop computer (Figure 1). The camera could be controlled by the computer through a time-lapse photography program (PSRemote, Breeze Systems, UK). The program was used to make the camera take a continuous stream of time-lapse photos at any desired interval and length of time. All photos taken by the camera were directly transferred to and saved on the computer.

### 2.4. Host Cues

The host cues were provided in the form of human breath, which produces an excited response in hungry bed bugs [20]. Breath was administered through a 1.98 m long plastic tube (1.27 cm inner diameter). One end of the tube was attached with Velcro (Velcro USA Inc., Manchester, NH) to an inner corner of the arena, pointing directly downward (Figure 1). For each replicate, the location of the tube was randomized to one of the four corners. The other end of the tube was positioned outside of the room. An experimenter at this end administered a steady supply of breaths during each replicate, by breathing normally into the tube. The same experimenter was used for all of the replicates. In previous studies, heat has been shown to be highly attractive [16,17,19]. In order to eliminate behavioral thermotaxis, the tubing was run through an ice bath. As a result, breath expelled from the tube was 1 °C–2 °C cooler than the surrounding air temperature, when measured directly beneath the tube, and unnoticeable when measured from the center of the arena.

### 2.5. Experimental Protocol

Experiments were performed in a controlled, room environment. The room stayed at a consistent 20.6 °C–21.7 °C during the experiments. All windows in the room were covered to block out the sunlight. A lamp, attached to the U-frame and covered with a Roscolux gel filter Light Red (#26) (Rosco Laboratories Inc., Stamford, CT), was used to illuminate the arena (Figure 1). Red light has been shown to produce no photonegative effects on bed bug behavior [18]. Thus, clear pictures could be taken, without the use of a camera flash. For each replicate, a small piece of white index card (1.91 × 0.95 cm) was cut, folded in half, and then dropped into a shell vial (4 mL). Five, adult bugs of the same sex were removed from the glass container and placed into the shell vial. The bugs and their harborage were kept inside of the vial for at least an hour, before being transferred to the arena. At this point, the shell vial was slowly inverted on top of the center point in the arena. The paper harborage and bed bugs slide out into the arena, but still remained trapped inside the inverted shell vial. After a few minutes, the shell vial was removed and an inverted Petri dish (5.08 cm diameter) was placed over the bugs. The bugs were allowed to acclimate beneath the Petri dish for an hour. The Petri dish was then removed, the experimenter began breathing into the tube, and the time-lapse system began taking pictures of the bugs over a 10 minute period. Working at an interval of 5 seconds between every shot, the camera captured 120 pictures during each replicate. Four replicates were completed for each sex in the presence and absence of olfactory cues, for a total of four treatments. Between replicates, the windows were opened and the room was vented.

### 2.6. Analysis

All of the pictures taken were processed using a custom designed program in MATLAB (The MathWorks, Natick, CA). The program expressed the bug's location in the picture as a polar coordinate, classified by a distance and an angle. Distance was the length measured from the center of the container to the bug's location. Angle was the measured degrees of difference between two imaginary lines pointing out from the center of the container. The first line was the 0° axis, which pointed towards the corner with tube. The second line was pointed towards the bug's location. A polar coordinate was gathered for all five bugs in all of the pictures. Each bug was individually tracked through all 120 photos of the replicate so that data from a bug in one picture could be linked with data from that same bug in all other pictures. Thus, the movement of each bug was described by 120 polar coordinates. Ten walking-path parameters (one circular and nine linear), derived from these coordinates, were used to describe the bugs' movement.

Circular statistics software Oriana 3.0 (Kovach Computing Services, Anglesey, Wales) was used to determine the single circular walking-path parameter, orientation (°). Using the coordinates, the mean vector (α) and length of the mean vector (r) were computed for each bug. α ranges from 0 to 360°, and indicates the average orientation that the bug was positioned relative to the arena's center. Alternatively, r ranges from 0 to 1, with 0 indicating an undefined path to α and 1 a straight path to α. Weighing α against r produced the weighted mean vector (α_w_), which ranges from 0 to 360°, and gives values further from the center more weight. α_w_ was used to describe the orientation of each bug during the experiment. For each treatment group, the average α_w_ of all bugs was computed. The Rayleigh test [21] was applied to each treatment group to test whether the orientation was circularly uniform (P > 0.05). If the Rayleigh test showed significant deviation from circular uniformity (P < 0.05), the V-test [21] was applied to assess if the orientation was statistically different from the direction of the tube (0°).

The polar coordinates were converted to Cartesian coordinates (x, y), and then used to compute the eight linear walking-path parameters describing the bug's movement: net displacement (cm), tortuosity, mean speed (cm/s), number of stops, walking time (%), mean distance from harborage (cm), mean distance to tube (cm), and time off harborage (s). The tortuosity is an index ranging from 0 and 1, with values close to 0 indicating a meandering path and values close to 1 indicating a straighter path. The linear walking-path parameters were computed using the statistical program R [22]. For each treatment group, the linear parameters were analyzed using a one-way analysis of variance (ANOVA) [23]. Total distance, tortuosity, average speed, and number of stops required square-root transformation; for clarity, the transformation values were only used for calculations of significance. When computing the linear walking-path parameters, movement was defined as a distance of 4 mm or more between successive coordinates, the body length of a bed bug. Any distance less than 4 mm was defined as not having moved. Additionally, the harborage area was defined as the circular region (4 cm diameter) that surrounded the note card at the center of the arena. Movement outside of this circle was considered to be off the harborage. Thus, for all of the linear walking-path parameters, data collected from inside of the circular region was discarded.

## 3. Results

**Figure 2:**
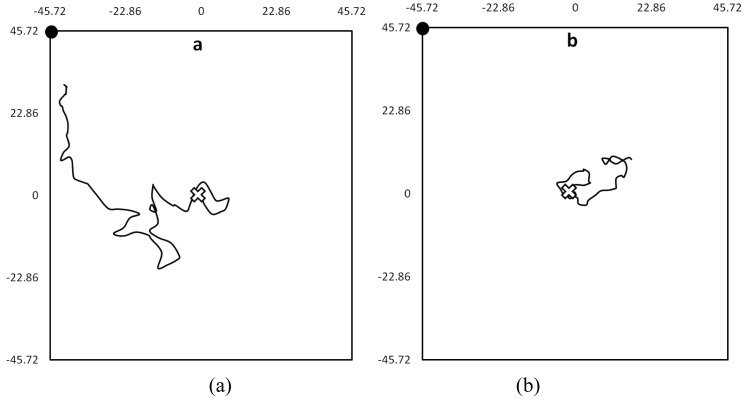
Sample walking-path of one bed bug, within the experimental arena (91.44 × 91.44 cm), demonstrating characteristic host-seeking behavior in the (**a**) presence and (**b**) absence of host breath. Tracks began (*white cross*) at the harborage in the center of the arena. In both instances, the breathing tube (*black dot*) was located in the upper left corner of the arena. The vertical and horizontal axes are measured in centimeters.

When the coordinates were connected in sequence, a series of walking-paths were produced, describing the bed bugs' movement. Walking-paths could best be described as meandering movement with frequent changes in direction. For example, (Figure 2) demonstrates the difference in walking-paths between bed bugs in the presence and absence of olfactory cues. Unapparent from these walking-paths were the short but frequent periods of motionlessness that interrupted longer periods of movement. While a majority moved away from the harborage area during the experiments, some bugs only moved inside of the harborage area, while others didn't move at all.

**Figure 3:**
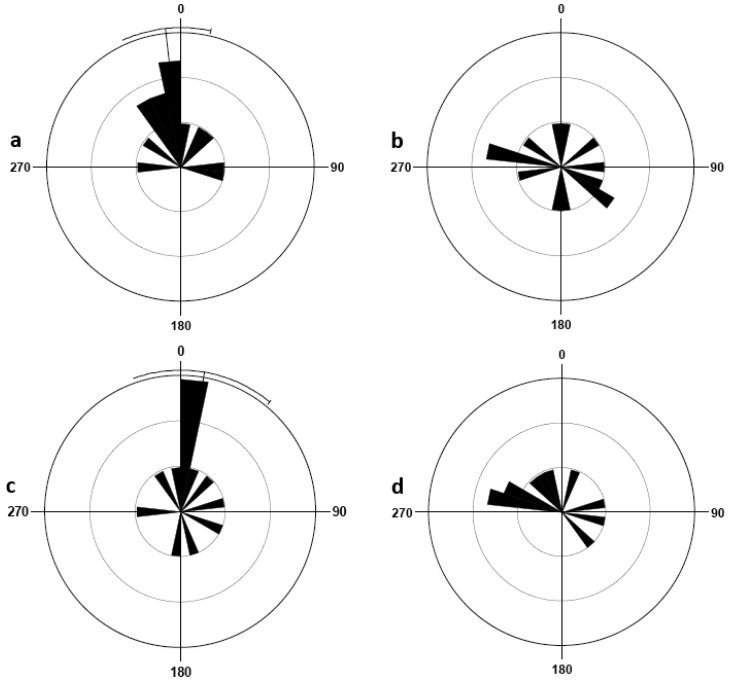
Orientation response of bed bugs in the four treatment groups: females in the presence of host breath (a), females in the absence of host breath (b), males in the presence of host breath (c), males in the absence of host breath (d). The rose diagram is a circular histogram, measured in degrees, progressing clockwise from the location of the breathing tube at 0°. Dark bars radiating from the center outward indicate the weighted mean vector, α_W_, of the bugs, with longer bar indicating a higher frequency of bugs and shorter bars a lower frequency of bugs. If Rayleigh test showed significant deviation from circular uniformity (P < 0.05), then the average α_W_ (*white line*) of the treatment group is shown with a 95% confidence interval.

In the presence of olfactory cues, male bed bugs on the central harborage were able to detect and then orient themselves in the direction of the tube (Rayleigh test, P = 0.002; V-test, P = 0.0002). Females demonstrated similar orientation behavior in the presence of olfactory cues (Rayleigh test, P = 0.000002; V-test, P = 0.0000002). Bed bugs in the absence of these olfactory cues showed a very different orientation response. Male orientation throughout the container was uniformly distributed (Rayleigh test, P = 0.099). Likewise, females in the absence of olfactory cues adopted a uniformly distributed orientation (Rayleigh test, P = 0.994). The rose diagrams (Figure 3) illustrate the orientation of bed bugs in each of the four treatments.

There were no significant differences among genders or attractant status for tortuosity and the number of stops. However, all remaining variables show differences between attractant and control for all genders and gender is not significant either by itself or as an interaction with the attractant status.

Among males, the attractant group showed a significant increase in five of the eight linear walking path parameters, as compared to the control (Table 1). The difference between the two groups, for the mean of the five parameters, were as follows: net displacement: 25.22 cm (D.F. = 73, t = 4.71, P = 0.00007); mean speed: 0.285 cm/s (D.F. = 73, t = 4.66, P = 0.00008); walking time: 42.52% (D.F. = 73, t = 4.30, P = 0.0003); mean distance from harborage: 18.63 cm (D.F. = 73, t = 4.35, P = 0.0003); time off harborage: 275.75 s (D.F. = 73, t = 4.38, P = 0.0002). There was a significant decrease compared to the control for mean distance to the tube: -12.39 cm (D.F. = 73, t = -3.02, P = 0.018).

Among females, there was a significant increase compared to the control for five of the eight linear walking-path parameters (Table 1). The difference of the five parameter means were as follows: net displacement: 28.34 cm (D.F. = 73, t = 5.29, P = 0.000007); mean speed: 0.305 cm/s (D.F. = 73, t = 4.22, P = 0.0004); walking time: 31.85% (D.F. = 73, t = 3.22, P = 0.01); mean distance from harborage: 18.89 cm (D.F. = 73, t = 4.41, P = 0.0002); time off harborage: 293.5 s (D.F. = 73, t = 4.66, P = 0.00008). Mean distance to tube: -21.78 cm (D.F. = 73, t = -5.31, P = 0.000007) showed a significant decrease compared to the control.

## 4. Discussion

In the natural world, a large number of exogenous factors such as light, temperature, time of day, and host stimuli may influence the observed activity of bed bugs [5,14,19]. Just as it is important to consider exogenous factors, likewise, endogenous factors such as gender, life-stage, and nutritional state can equally influence movement behavior [18,24,25]. Among insects, such factors influence the demonstrated searching behavior for resources items: food, mates, and shelter [26]. Outside of a controlled environment, a natural blending of factors would cause searching behavior patterns to overlap and make it difficult to differentiate between them. For instance, activity in recently fed, bed bug males might be an effort to find a potential host, or a strategy to improve mating opportunities [14]. However, under the conditions of this experiment, the bed bug's movement can be attributed predominantly to host searching behavior.

Although the males and females were separated, mate searching would have played a negligible role in the movement behavior of the bed bugs. Bed bug copulation typically occurs after the acquisition of a blood meal [27]. Since all insects had been starved, the influence of mate-seeking behavior on the movements of segregated males and females was most likely negligible. Additionally, at no point during the experiments did males attempt sexual mounting, a gender-indiscriminate action performed by males initiating copulation [28]. Searching behavior for shelter would also have been a negligible influence. Thigmotactic affinity [6] and a preference for previously exposed areas [29] compel the formation of bed bug aggregations within refuge sites [1]. The bed bugs had been placed on the harborage well before the start of each test. We believe that the folded note card in the center of the arena was therefore sufficient enough to discourage shelter-seeking behavior by the insects. In the absence of a central harborage, bed bug behavioral patterns could have been reflective of both host and harborage-seeking. Aside from the discouragement of behavior associated with these two possible resources, other factors such as light and feeding state would have encouraged behavior associated host searching. It is known that aggregation behavior decreases as the bug's starvation increases [1]. As such, bed bug movement during this study can therefore best be attributed to host-seeking behavior.

The presence or absence of host breath produced host-seeking behavior that was either attractant driven or non-attractant driven. In the control treatments, the majority of males (86%) and females (82%) never departed from the harborage area. Alternatively, breath exposed treatments had a smaller proportion of males (19%) and females (13%) remain. Thus, in the absence of host cues, the experimental harborage likely provided sufficient chemical and thigmotactic conditions for most of the insects to remain immobile, while movement away could be attributed to non-attractant driven host-seeking. Although bugs from all treatments groups explored the surrounding arena, we believe this discrepancy highlights the importance of host stimuli as an exogenous factor motivating behavioral movement away from the harborage. In a starved state, bed bug aggregating cues can go largely ignored when presented against host cues.

In attractant driven host-seeking behavior, the initial orientation of the bugs was seldom directly towards the source of the breath. Though reported in an early behavioral study on the bed bug, it is important to reiterate the fact that host-seeking bed bug's host movement path can best be characterized by a tortuous meander, especially in the presence of host stimuli [19]. Strategically, a spiraling search improves the insect's chances of encountering the desired resource or other related cues in its surroundings [26]. This response to host cues and seemingly random movement characterizes the behavioral patterns of parasites that must actively search for a host in the environment, not the type that would lie in ambush for the host to come to them [30]. Rather than wait for a host to return, travel into other rooms or apartments would seem the likely response of starved bed bugs. This would support previous findings that bed bugs have been found moving from infested apartments to neighboring apartments, via the hallways [10,17].

Also, when exposed to breath from a human host, bed bugs altered their host-seeking behavior from localized to long range searching, indicated by the increase in net displacement and mean distance from the harborage. In obligatory hematophagous insects, like the bed bug, searching may be a double-edged sword, increasing the chance of locating a host, but also depleting limited energy stores [5,24]. Thus, resource specific cues would only stimulate localized searching in cases where it would be advantageous to thoroughly explore the immediate area for a host [26]. This is likely an indication as to how this specific host cue fits into the entire host-seeking pathway of the insect. Olfactory cues, such as carbon dioxide, are important in the long-distance attraction in the Tsetse fly, before other host stimuli play a role [31]. We would therefore speculate that the lack of interest in exploring the immediate environment, suggests the importance of human breath as a long range cue, under natural conditions. In long range attraction, the existence of other host stimuli and its possible synergetic effect with host breath is not completely understood and therefore demands future research. A more tortuous movement pattern would also indicate localized searching behavior [26]; however, there was no significant difference in the tortuosity of any of the four treatments. It is conceivable that a larger environment, longer testing period, or more frequent photographs could have magnified differences in tortuosity between the groups.

Animals in search of resources may exhibit stop-start searching behavior [32]. During stop-start searching, an insect periodically halts movement, while scanning the environment for resources or sensory information [26]. We observed the majority of the active bed bugs stop for short periods during their movement paths, resuming their movement usually after a shift in orientation. In the case of the host-seeking bed bug, a repeated sampling of the immediate surroundings would ensure that host cues in the environment don't go overlooked. Additionally, repeated sampling would also theoretically be a favorable searching behavior for bed bugs even after the initial detection of a host. Stimulated by host odor, the host-seeking bug could continually adapt its orientation as necessary, based on periodic, sensory input indicating the direction and intensity of the cue, eventually leading directly to the host. The blood-feeding triatomine *Rhodnius prolixious* in the presence of chemostimuli on a servosphere will similarly perform antennal scanning, during pauses in movement [33]. The similar frequency of stops by groups in the presence and absence of host breath suggests that stop-start searching plays a principal role in both attractant mediated host-seeking and non-mediated host-seeking. In the former, the behavior assists in the orientation of the bugs towards the odor source. In the later, the behavior helps in the detection of potential host cues.

Had the human breath only promoted an activation response, the difference in behavior between bugs in the presence of the attractants and bugs in the absence of the attractant would have resulted in only heightened levels of movement activity among the exposed insects. Such host-seeking behavior would still have been advantageous, improving the likelihood that the searching insect might come across the desired resource [26]. As expected, higher activity across nearly all of the linear-walking path parameters was observed for males and females when exposed to the specific host odors in human breath. However, the differences in orientation between the attractant and control groups suggests that the long range chemical components of human breath not only activates the bed bug's host-seeking response, but also acts as a direct attractant leading the insect almost directly from harborage to host over a short period of time. The insect's rapid and direct response to a human, even to one a fair distance from the harborage, is a testament to the efficiency with which bed bugs can spread throughout all areas of a room, while still parasitizing a host that may not be in close proximity [34].

This experiment fails to indicate over exactly what size distance the bed bug may detect a host from breath alone, which is information crucial in understanding the factors driving host-seeking behavior and movement. The results of our study don't negate the importance of other host cues, such as body heat, in the bug's host-seeking pathway. The experimental design only allowed for a study of the host-seeking behavior from the harborage through the environment. For example, heat and proper texture are both important conditions that must be present before feeding commences [19], but were not of consideration here. It should also be noted that the bed bugs used in our experiment came from a strain fed for more than 30 years, through direct contact on Dr. Harlan. Without the need to employ long range host-seeking behavior, the sensory and motor capabilities of this domestic strain may have diminished in intensity in comparison to a wild strain, though they would likely both exhibit the same general, behavioral characteristics.

## 5. Conclusion

Male and female bed bugs demonstrate a non differential host-seeking response both in the presence and absence of human breath. Additionally, the altered orientation and movement parameters of both sexes' suggest that such long-range host odors promote behavioral chemotaxis. Using a similar experimental design, future work on host-seeking could compare the bed bugs' behavioral reaction to true host odor and chemical baits.

## Figures and Tables

**Table 1:** Mean, standard error, and total number of bugs that moved (*N*) for the eight linear walking-path parameters of *Cimex lectularius.* Means in the same column with the same letters are not significantly different from each other at the 5% level of significance using Tukey's test. (Maxiun width of table: 29.7-1.75*2=26.2)

**Treatment Group**		***N***	**Net Displacement (cm)**	**Tortuosity**	**Mean Speed (cm/s)**	**Number of Stops**	**Mean Distance from Harborage (cm)**	**Walking Time (%)**	**Mean Distance to Tube (cm)**	**Time off Harborage (s)**
Female	Attractant	20	40.2a ±4.3	0.306a ±0.054	0.438a ±0.074	5.20a ± 1.22	30.0a ±3.1	57.3a ±8.2	44.5a ±3.0	439a ±56
	Control	20	11.9b ±4.3	0.261a ±0.078	0.133b ±0.030	2.72a ±0.75	11.1b ±3.1	25.5b ±8.2	66.3b ±3.0	146b ± 56
Male	Attractant	20	34.2a ±4.3	0.233a ±0.048	0.384a ±0.068	5.12a ± 1.20	23.5a ±3.1	59.7a ±8.2	51.2a ±3.0	397a ±56
	Control	20	9.0b ±4.3	0.409a ±0.086	0.099b ± 0.020	2.98a ±0.81	4.9b ±3.1	17.2b ±8.2	63.5b ±3.0	121b ±56
